# Marine natural compounds can be efficient toward aflatoxigenic *Aspergillus flavus *strain

**DOI:** 10.1186/1753-6561-8-S4-P114

**Published:** 2014-10-01

**Authors:** Yasmim Dantas Crivelenti, Cyntia AM Arevabini, Mariana H De Abreu, Tamires Aparecida Bitencourt, Thaís B Mesquita, Bruna AM Cantelli, Mario FC Santos, Roberto GS Berlinck, Eduardo Hajdu, Renê de O Beleboni, Mozart Marins, Ana Lúcia Fachin

**Affiliations:** 1 Unidade de Biotecnologia, Universidade de Ribeirão Preto, CEP 14096-900, Ribeirão Preto, SP, Brazil; 2 Instituto de Química de São Carlos, Universidade de São Paulo, CP 780, CEP 13560-970, São Carlos, SP, Brazil; 3 Museu Nacional, Universidade Federal do Rio de Janeiro, Quinta da Boa Vista, s/n, Rio de Janeiro, RJ, Brazil

## Background

The presence of high levels of aflatoxin is a serious problem to the production of raw peanuts and peanut crumbs. The high incidence of aflatoxin in peanuts in our country is mainly due to problems in primary production. High humidity and temperature conditions increase the likelihood of *Aspergillus *development and aflatoxins production, which is worsened during rainy weather [1]. Aflatoxins may remain in the food after the death of fungus without visible alterations [2]. The effects of aflatoxins on human and animal health, besides resistance of fungi to conventional antifungal agents has motived the search for new inhibitors. The research of marine natural products from sponges has been considered as a promising source for the development of new antifungal agents in order to discover compounds more effective and less toxic [3]. The objective of this study was to evaluate the antifungal activity of 21 marine natural compounds toward an aflatoxigenic *A.flavus *ATCC strains.

## Methods

Aflatoxin producing (CCT 7836) and non producing (ATCC9643) *A. flavus *strains were purchased from Fundação André Tosello, Campinas, Brazil and were kept in Sabouraud media at 35 ºC. The minimum inhibitory concentration (MIC) of 21 sponge marine natural products, identified as SM1 to SM21, toward the two strains of *A.flavus *was determined by microdilution assay in 96-well plates using RPMI medium according to the protocol NCCLS M-38 [4] for 7 days at 37C, using commercial antifungal cercobin as control.

## Results and conclusions

The marine product SM5 (CIM = 3.9 µg/mL) was more effective than cercobin (CIM = 7.8 µg/mL) against *A. flavus *control aflatoxin producer strain. Moreover, marine product SM5 (MIC = 1.9 µg/mL) was also a more effective antifungal agent than cercobin (MIC = 3.9 µg/mL) against the *A. flavus *ATCC strain. The findings suggest that natural marine products are a promising source of new molecules for the development of antifungal compounds against aflatoxigenic fungus that affect public health and the food production.

## References

[B1] Hedayati MT Pasqualotto AC Warn PA Bowyer P Denning DW Aspergillus flavus: human pathogen, allergen and mycotoxin producer Microbiology 2007 153 1677 10.1099/mic.0.2007/007641-0 17526826

[B2] Yu J Cleveland TE Nierman WC Bennett JW Aspergillus flavus genomics: gateway to human and animal health, food safety, and crop resistance to diseases Revista Iberoamerica de Micologia 2005 22 194 10.1016/S1130-1406(05)70043-7 16499411

[B3] Mayer AM Rodriguez AD Berlinck RG Fusetani N Marine pharmacology in 2007-8: Marine compounds with antibacterial, anticoagulant, antifungal, anti-inflammatory, antimalarial, antiprotozoal, antituberculosis, and antiviral activities; affecting the immune and nervous system, and other miscellaneous mechanisms of action Comparative biochemistry and physiology Toxicology & pharmacology : CBP 2011 153 191 222 10.1016/j.cbpc.2010.08.008 20826228 PMC7110230

[B4] Reference method for broth dilution antifungal susceptibility testing of conidium-forming filamentous fungi Proposed standard NCCLS document M38-A 2002 National Committee for Clinical Laboratory Standards, Wayne National Committee for Clinical Laboratory Standards

